# Optimization of ultrasound-assisted extraction of poly-phenols from *Ajuga ciliata Bunge* and evaluation of antioxidant activities *in vitro*

**DOI:** 10.1016/j.heliyon.2019.e02733

**Published:** 2019-11-01

**Authors:** Yanfei Zhang, Haitong Tang, Yuchuan Zheng, Jinzhu Li, Le Pan

**Affiliations:** a School of Information Engineering, Huangshan University, Tunxi, 245041, PR China; b School of Chemistry & Chemical Engineering, Huangshan University, Tunxi, 245041, PR China

**Keywords:** Analytical chemistry, Food science, *Ajuga ciliata Bunge*, Poly-phenols, Ultrasonic, RSM, FTIR

## Abstract

Effective extraction of natural antioxidants from cheap plant sources is still a problem. In this paper, an excellent method of ultrasound-assisted extraction of phenolic compounds from *Ajuga ciliata Bunge* was studied. The effects of four factors including ethanol volume fraction, ultrasonic time, ultrasonic temperature and material liquid ratio were discussed. After single factor experiments had been investigated, a 4-factor, 3-level Box-Behnken design experiment was used to obtain the model optimum conditions, which are shown as follows: ethanol volume fraction of 41%, liquid-solid ratio of 35:1 mL/g, ultrasonic temperature of 60 °C and ultrasonic time of 50 min. Under these conditions, the experimental productivity is 3.552 mg/g. The spectra of Fourier infrared and energy dispersive X-ray suggest that phenolic compounds exist in the extracts. Besides, free radical scavenging potentials of superoxide anion, hydroxyl and DPPH were measured to evaluate their antioxidant properties. This study proves that the ultrasonic-assisted extraction technique can extract phenolic compounds with antioxidant capacity from *Ajuga ciliata Bunge*.

## 1 Introduction

Biomacromolecules including nucleic acids, proteins, carbohydrates, lipids, are important parts of the organism, which not only have large molecular weight, complex structure but also have many biological functions. Once biomacromolecules are affected by abnormal factors, there will be some serious consequences. For example, oxidative damage to biological macromolecules causes many chronic diseases, such as cardiovascular disease, cancer and aging [1,2]. Some substances are called antioxidants because they have the function of resisting the oxidation of biological macromolecules and attenuating human tissue and cell damage. Natural antioxidants are a class of antioxidants that have great potential in the food, herbal, and feed industries. They can be extracted from inexpensive natural organisms, including polyphenols, flavonoids, polysaccharides and so on [3,4].

In order to effectively obtain natural antioxidants, it is important to study the extraction methods of natural organisms. Initially, the extraction of natural antioxidants was carried out by hot solvent extraction [5,6]. Subsequently, the extraction of natural antioxidants was carried out another method named Soxhlet extraction，which can overcome some shortcomings of the hot solvent extraction method. However, both two traditional methods are not efficient techniques due to its long extraction time, energy-wasting and low extract yield. Recently, some techniques have been employed to overcome these drawbacks, such as microwave-assisted extraction technique [7,8], ultrasound-assisted extraction (UAE) technique [9] and supercritical fluid extraction technique [10,11]. Among them, UAE technology for natural antioxidant extraction has great potential in the herbal and food industries [12,13], because it shows many advantages such as short extraction time, low solvent consumption and low energy consumption [14,15]. Therefore, UAE has become an emerging extraction technology of poly-phenols [16,17], flavonoids [18], and polysaccharides [19,20]. Poly-phenols are one of the most common natural antioxidants and are a class of compounds containing multiple phenolic groups in natural products that can be extracted from natural sources and studied extensively. Many researchers have studied the effective extraction of poly-phenols from natural resources and evaluation of their antioxidant activities [21,22]. It is very important to optimize the extraction factors to obtain the maximum poly-phenols content [23].

*Ajuga ciliata Bunge* is a perennial herb of *Ajuga* that is inexpensive, easy to obtain, and widely distributed in Europe, North America, China, Japan and other countries [24]. The plants of *Ajuga ciliata Bunge* can be used as medicinal products in China for hundreds of years [25–27] because it has good effects on treating some symptoms of upper respiratory tract infection, tonsillitis, bronchus, high blood pressure, burns, scalds and so on [27,28]. Many researchers believed that the plants of *Ajuga ciliata Bunge* have many valuable constituents, including ecdysterone, cyasterone, ajugasterone B, ajugasterone C, ajugalactone, saponins, alkaloids, and poly-phenols, and these constituents show various biological activities [29]. Among these biological components, poly-phenols can prevent these chronic diseases and attract the attention of many scientists.

In this paper, the UAE method was used to extract poly-phenols from *Ajuga ciliata Bunge*. To obtain the best extraction conditions of UAE, response surface method (RSM) was hired. Through empirical methods, optimization of extraction process factors requires a lot of time. RSM is a very effective statistical method, which is used to optimize process variables and is widely used to optimize the extraction process parameters of poly-phenols, because it can reduce the number of trials [30,31]. To our best knowledge, there are no reports on the extraction of polyphenols from *Ajuga ciliata Bunge* using UAE technology by RSM. The current work is of great value compared to previous extraction studies of *Ajuga ciliata Bunge*. Firstly, fast and energy-saving ultrasonic extraction was employed instead of traditional solvent extraction. Secondly, advanced RSM was used to replace the traditional orthogonal design to optimize the extraction process. Thirdly, the technologies of FTIR and energy dispersive X-ray (EDX) were used to characterize poly-phenols extracts. Lastly, the poly-phenols of *Ajuga ciliata Bung*e (PAB) have significant hydroxyl radical scavenging ability, superoxide anion radical scavenging ability and DPPH free radical scavenging ability, which indicate PAB can be utilized as natural antioxidants. This study has certain guiding significance for the extraction and application of poly-phenols from *Ajuga ciliata Bunge*.

## 2 Materials and methods

### 2.1 Materials and reagents

*Ajuga ciliata Bunge* was purchased from the local market in Huangshan City. After being dried for 80 h at 41 °C in a hot air oven, the dried *Ajuga ciliata Bunge* was ground and conceded through a 100-mesh sieve, sealed in plastic bottle, and stored in the refrigerator at 4 °C. 1,1-Diphenyl-2-picryl-hydrazyl (DPPH), Tris (hydroxymethyl)methyl aminomethane (THAM), ascorbic acid (Vc), salicylic acid (SAA), Gallic acid, phenanthroline, CH_3_CH_2_OH, ethanol, FeSO_4_, Na_2_HPO_4_, NaH_2_PO_4_, Li_2_SO_4_, Na_2_MoO_4_, Na_2_WO_4_, HCl, H_3_PO_4_, Na_2_CO_3_, H_2_O_2_ and Br_2_ were of analytical grade and purchased from the Sinopharm Chemical Reagent Co. Ltd, China.

### 2.2 UAE of poly-phenols from *Ajuga ciliate Bunge*

The poly-phenols from *Ajuga ciliata Bunge* by UAE was performed in a KQ-500E ultrasonic bath (Yuhua, China), a rectangular container with transducers at a frequency of 28 kHz. In brief, 0.2000 g of the dried ground *Ajuga ciliata Bunge* plant was thoroughly mixed with 7 mL 40% ethanol and placed in a 25 mL glass tube. When the bottle was immersed in the ultrasonic bath, the liquid level in the glass tube was slightly lower than that in the ultrasonic cleaner to take advantage of the maximum ultrasonic energy. The ultrasonic power was fixed to low power of 90 W and the influence of other factors on the extraction rate of PAB were studied by single-factor design. Single-factor experiments were carried out in a designed liquid-solid (LS) ratio, ethanol volume concentration (EVC), ultrasonic temperature (UTe) and ultrasonic time (UTi) to extract poly-phenols. After the extraction tests, the ultrasonically extracted slurry was filtered with medium speed quantitative filter paper, and then filter liquor was transferred to centrifugal pipe and centrifuged at 4000 rpm for 5 min, the supernatant obtained was collected as poly-phenol of *Ajuga ciliata Bunge* (PAB) in a volume bottle.

### 2.3 Determination of PAB content

Content of PAB was measured using Folin–Ciocalteu reagent method [32]. The Folin–Ciocalteu reagent was prepared by us in our laboratory. The main determination steps of PAB content were as follows: 0.1 mL PAB was mixed with 0.5 mL Folin–Ciocalteu reagent and 8.4 mL water. After incubated at 25 °C for 6 min, 1.0 mL 20wt% Na_2_CO_3_ was added and the absorbance was measured at 760 nm.

### 2.4 Characterizes of PAB

The supernatant was poured into a rotary evaporative bottle, and ethanol was recovered by rotary evaporation at 45 °C under vacuum. The concentrated solution was dried to constant weight at 50 °C to obtain PAB. The functional groups of PAB were characterized by Fourier transform infrared spectroscopy using a FITR-850 spectrophotometer (Gangdong, China) with KBr technique. The surface morphologies of PAB were observed by a scanning electron microscope (SEM) (S–3400N, Japan) equipped with EDX.

### 2.5 Statistical analysis [33,34]

Each single-factor experiment was carried out three times, the optimum technological conditions for extracting poly-phenols from *Ajuga ciliata Bunge* were established by RSM [35,36] and the results of PAB were statistically analyzed by Design-Expert Software.

### 2.6 Antioxidant activity

#### 2.6.1 Hydroxyl radical (HO·) scavenging capacity [37,38]

Firstly, 2.0 mL of each PAB was mixed with 2.0 mL 6 mmol/L FeSO_4_, 2.0 mL 6 mmol/L SAA-ethanol solution, 2 mL 30% H_2_O_2_. Then, the solution mixture was adjusted to 12 mL and incubated at 25 °C for 30 min in dark. Finally, the absorbance was read at 510 nm and designated as A_1_. Another group of solution was measured using 2 mL water in place of 2 mL H_2_O_2_. The absorbance was specified as A_10_. Additionally, the absorbance of the background solution was designated as A_0_. The formula for HO**·** scavenging capacity of PAB is as follows:

(1) $$\text{HO}•\text{scavenging ​activity}\left(\text{\%}\right)=\frac{A_{0}-\left(A_{1}-A_{10}\right)}{A_{0}}\times 100\%$$

#### 2.6.2 Superoxide anion radical (O_2_^-^·) scavenging activity [39]

1.5 mL of each PAB was mixed with 1.8 mL THAM-HCl (50 mmol/L, *p*H 8.2) and 1.0 mL pyrogallic acid (1 mmol/L). Then, they were incubated with 25 °C for 12 min and their absorbencies at 320 nm were denoted as A_2_. The absorbance of the background solution was expressed as A_20_. The formula is as follows:

(2) $${{\text{O}}_{2}}^{-}•\text{scavenging ​activity}\left(\text{\%}\right)=\frac{A_{20}-A_{2}}{A_{20}}\times 100\%$$

#### 2.6.3 DPPH radical scavenging capacity [40,41]

5.0 mL of each PAB was mixed with 1.0 mL 0.2 mmol/L DPPH and incubated in the dark at 25 °C for 30 min. The absorbance was read at 510 nm by UV-visible spectroscopy and designated as A_3_. The absorbance of the background solution was denoted as A_x_. The absorbance of PAB solution without added DPPH solution was read at 510 nm designated as A_30_. The formula is as follows:

(3) $$\text{DPPH ​}•\text{ ​scavenging ​capacity}\left(\text{\%}\right)=\left(1-\frac{A_{3}-A_{30}}{A_{x}}\right)\times 100\%$$

## 3 Results and discussions

### 3.1 Single-factor experiments

**Fig. 1:**
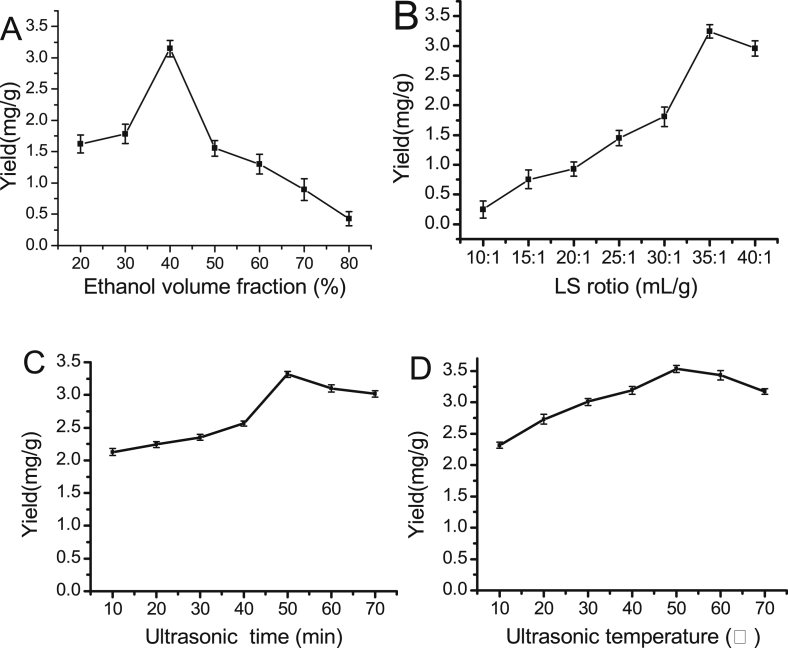
The average effect of the EVC(A), LS ratio(B), UTe(C) and UTi(D) (n = 5).

Extraction experiments of PAB are carried out at various LS ratio, EVC, UTe, and UTi. The results are shown in Fig. 1, the optimal conditions for each single-factor can observed as follows: EVC of 40%, LS ratio of 35:1 mL/g, UTi of 50 min and UTe of 50 °C. Depending on the optimal conditions of each single factor, the response values (LS ratios, X_1_; EVC, X_2_; UTe, X_3_ and UTi, X_4_) for yields of PAB is shown in Table 1.

**Table 1:** Selected factors and levels.

Factors	Symbol	Levels		
LS ratio/(mL/g)	X_1_	-1 (30:1)	0 (35:1)	1 (40:1)
EVC/%	X_2_	-1 (30)	0 (40)	1 (50)
UTe/°C	X_3_	-1 (40)	0 (50)	1 (60)
UTi/min	X_4_	-1 (40)	0 (50)	1 (60)

### 3.2 Fitting the model

Depending on the selected factors and levels are shown in Table 1, 29 experiments of poly-phenols extraction from *Ajuga ciliata Bunge* are performed and the obtained results are listed in Table 2. Box-Behnken design (BBD) is employed to estimate the effect of response values (LS ratios, X_1_; EVC, X_2_; UTe, X_3_ and UTi, X_4_) for yields of PAB and the results of PAB extract experiment by UAE are shown in Table 2. The resulting model in terms of code to predict the optimal point of extraction are presented below in Eq. (5)

(5) $$\text{Y ​}{=\text{3.52}+0.14\text{X}}_{1}{+0.12\text{X}}_{2}-{0.11\text{X}}_{3}{+0.021\text{X}}_{4}{+0.047\text{X}}_{1}{\text{X}}_{2}+0.14{\text{ ​X}}_{1}{\text{X}}_{3}{+0.075\text{X}}_{1}{\text{X}}_{4}{+0.20\text{X}}_{2}{\text{X}}_{3}{+0.088\text{X}}_{2}{\text{X}}_{4}{+0.22\text{X}}_{3}{\text{X}}_{4}-0.95{{\text{X}}_{1}}^{2}-1.06{{\text{X}}_{2}}^{2}-0.91{{\text{X}}_{3}}^{2}-0.87{{\text{X}}_{4}}^{2}$$

**Table 2:** BBD and the response values for yields of PAB.

No	X_1_/(mL/g)	X_2_/%	X_3_/°C	X_4_/min	Y/(mg/g)
1	0	0	-1	-1	2.3499
2	-1	0	1	0	1.2470
3	0	-1	1	0	1.0408
4	0	1	-1	0	1.5641
5	-1	0	-1	0	1.5718
6	1	0	0	1	2.0057
7	1	1	0	0	1.8423
8	-1	0	0	1	1.5285
9	0	0	0	0	3.5024
10	0	0	1	1	1.8156
11	0	1	1	0	1.7998
12	1	0	1	0	1.8438
13	0	0	0	0	3.5426
14	0	1	0	1	1.7087
15	0	-1	0	1	1.2938
16	0	0	0	0	3.5206
17	-1	-1	0	0	1.5351
18	1	0	0	-1	1.6427
19	0	0	0	0	3.4908
20	0	1	0	-1	1.5185
21	1	-1	0	0	1.6250
22	-1	1	0	0	1.5630
23	0	-1	0	-1	1.4573
24	0	0	-1	1	1.8093
25	-1	0	0	-1	1.4650
26	0	0	1	-1	1.4793
27	1	0	-1	0	1.5987
28	0	0	0	0	3.5238
29	0	-1	-1	0	1.6039

ANOVA is used to evaluate the significance of the model and the results are presented in Table 3. The p-values of three linear terms (LS ratios, X1; EVC, X2; UTe, X3) all less than 0.05 (marked as “*” in Table 3) indicating that the extraction yield of PAB is significantly affected by these linear terms. The p-values of two interactive terms (X_2_X_3_, X_3_X_4_) are all less than 0.05 implies that the extraction yield of PAB is significantly affected by X_2_X_3_ and X_3_X_4_. The p-values of other interactive terms (X_1_X_2_, X_1_X_3_, X_1_X_4_, and X_2_X_4_) are more than 0.1, which indicates that these four interaction factors are not significant. The p-values of four quadratic terms (LS ratios, EVC, UTe, and UTi) are lower than 0.0001 (marked as “**” in Table 3), which suggests that the extraction yield of PAB is extremely significantly affected by those quadratic terms. The p-value of lack-of-fit is 0.0003, which shows that the applicability of the model can accurately predict variation.

**Table 3:** ANOVA for the fitted quadratic polynomial model.

Source	Degree of freedom	Sum of Squares	Mean square	F value	p-Value	Rem-arks
Model	16.05	14	1.10	41.10	<0.0001	**
X_1_	0.23	1	0.22	8.11	0.0129	*
X_2_	0.17	1	0.17	6.20	0.0260	*
X_3_	0.13	1	0.13	4.83	0.0453	*
X_4_	0.0052	1	0.0052	0.19	0.6735	
X_1_X_2_	0.0090	1	0.0090	0.32	0.5795	
X_1_X_3_	0.081	1	0.081	2.91	0.1100	
X_1_X_4_	0.022	1	0.021	0.80	0.3850	
X_2_ X_3_	0.16	1	0.16	5.72	0.0314	*
X_2_ X_4_	0.031	1	0.031	1.12	0.3076	
X_3_ X_4_	0.19	1	0.19	6.89	0.0200	*
X_1_^2^	5.80	1	5.80	207.99	<0.0001	**
X_2_^2^	7.20	1	7.29	261.42	<0.0001	**
X_3_^2^	5.42	1	5.42	194.19	<0.0001	**
X_4_^2^	4.91	1	4.91	176.03	<0.0001	**
Residua	0.39	14	0.028			
Lack of fit	0.39	10	0.039	96.46	0.0003	
Pure error	0.0016	4	0.0016			
Cor total	16.44	28				

R^2^ = 0.9762 Adj-R^2^ = 0.9525 Pred-R^2^ = 0.8636 C.V.% = 8.57%.

Notes: *means significant difference, ** means extremely significant difference.

The value of R^2^ ranges from 0 to 1. The greater the R^2^ value is, the more accurate the model is. When the R^2^ value is 1, the model is most accurate. As shown in Table 3, the value of R^2^ is high on 0.9762, which indicates the model is very accurate. R^2^ is influenced by the number of independent variables. To delete the influence of independent variables, Adj-R^2^ replaced R^2^. Adj-R^2^ is a modification of R^2^, it can help people better judge the pros and cons of the model [42]. The value of Adj-R^2^ is 0.9525, which indicates that the model is very accurate. The low C.V. % (8.57%) denotes that the experiments performed are reliable. These results report that the model could work well to predict the extraction effect of *Ajuga ciliata Bunge* extraction effectiveness.

### 3.3 Analysis of response surfaces

**Fig. 2:**
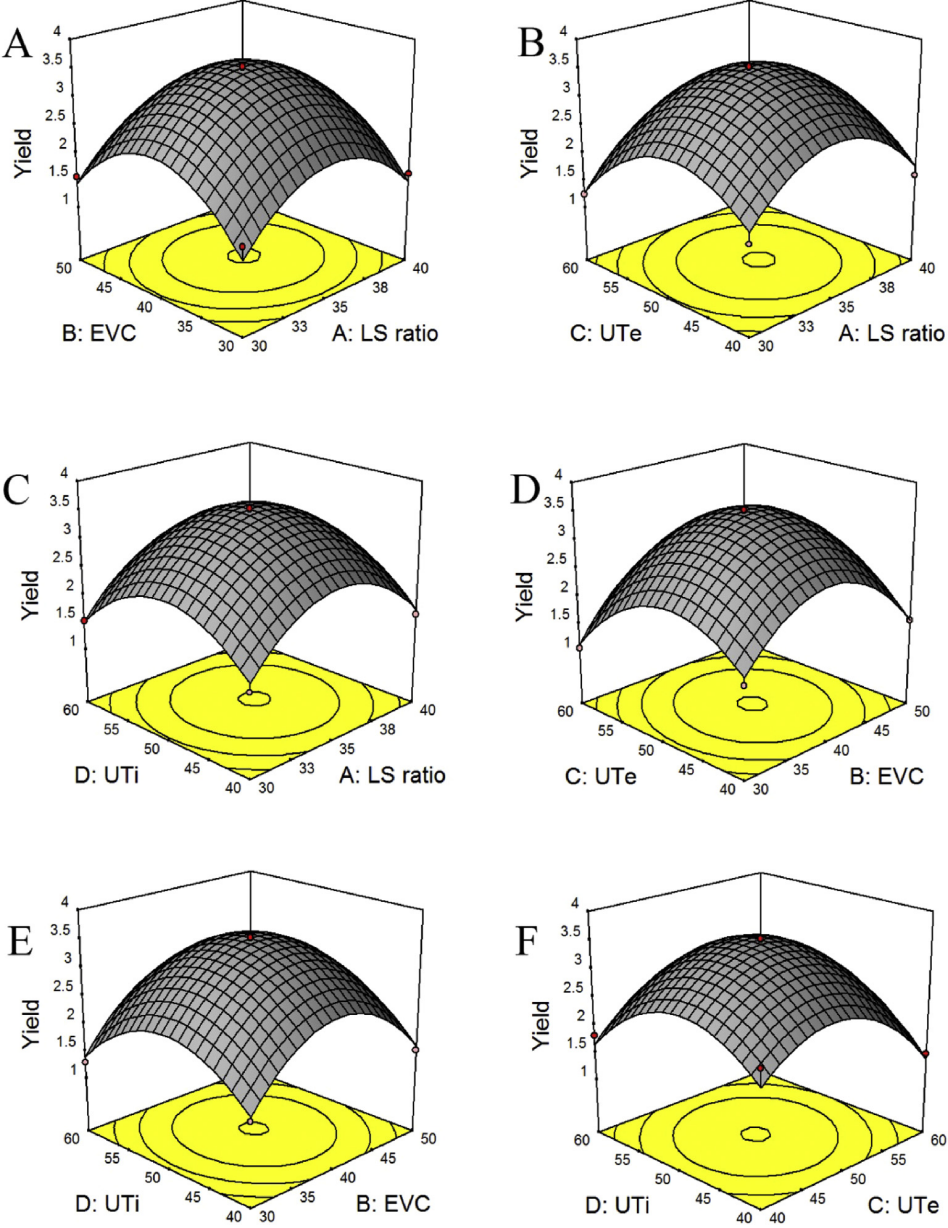
The interactive effect of the variables on *Ajuga ciliata* Bunge: (A)LS ratio versus EVC, (B)LS ratio versus UTe, (C)LS ratio versus UTi, (D)EVC versus UTe, (E)EVC versus UTi, (F)UTe versus UTi.

The 3D response surface is presented in Fig. 2. As shown in Fig. 2A, while keeping UTe at 50 °C and UTi at 50 min, the 3D response is generated as a function of LS ratio (30:1–40:1 mL/mg) and EVC (30–50%). It can be found that EVC has a strong effect on the yield of PAB, while the extraction time has only a limited effect. From Fig. 2B, when EVC and UTi are fixed, the PAB yield raises with increment the LS ratio and UTe. From Fig. 2C, when EVC and UTi are fixed, the effect of UTi on the response variables is greater than that of LS ratio. As present in Fig. 2D, when LS ratio and UTi are fixed at 0 levels, EVC pictures a strong influence on the yield of PAB, and UTe has a slight impact on the yield of PAB. According to Fig. 2E, when keeping LS ratio and UTi at 0 levels, EVC and UTe show a similar influence on the response variables. According to Fig. 2F, when LS ratio and UTe are kept at 0 levels, it can be found that UTi has a strong effect on the yield of PAB, while EVC has only a limited effect. Base on 3D response surface and the model, the optimal values of the selected variables can be obtained by Expert-Design software and the optimal conditions are as follows: EVC of 40.54%, LS ratio of 35:1 (mL/g), UTe of 59.55 °C, UTi of 50.13 min. Under optimal conditions, the model predicted a maximum yield of Y_max_ = 3.52531 mg/g.

### 3.4 The highest extraction rate of the experiment

According to the results of the model predicted，PAB extraction experiment was performed under those conditions：EVC of 41%, LS ratio of 35:1 (mL/g), UTe of 60 °C and UTi of 50 min the experimental maximum yield of Y_max_ = 3.55166 mg/g. The error between the predicted value and the experimental value is only 0.95%, which present that the model by BBD can work well.

### 3.5 Antioxidant activities

#### 3.5.1 HO· scavenging activity

**Fig. 3:**
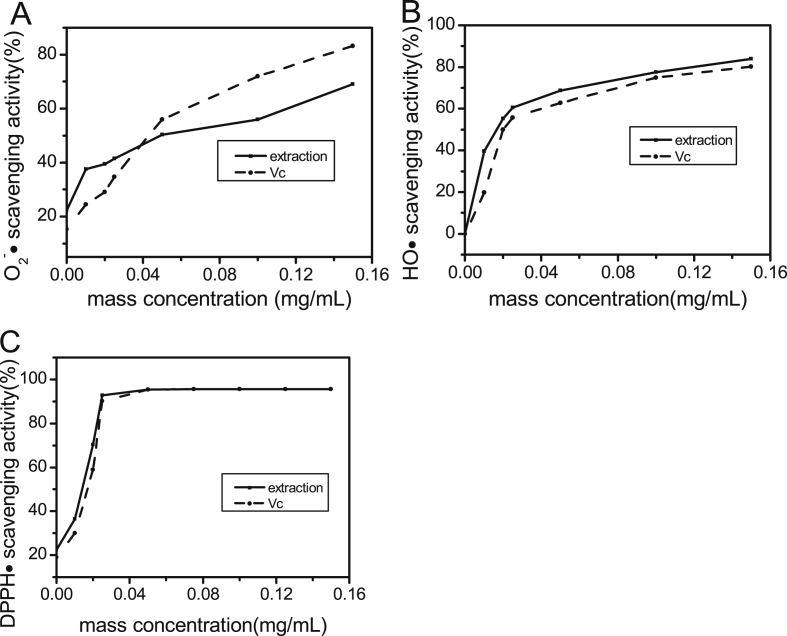
The antioxidant activities of PAB: (A)HO scavenging activity, (B)O_2_^-^·scavenging activity and (C)DPPH·scavenging activity.

As can be seen from Fig. 3A, when the concentration was increased from 0.00 to 0.16 mg/mL, the HO**·** scavenging activity of PAB increased rapidly from 0% to 83.81%. Compare to Vc, the activity of PAB is slightly stronger, indicating that poly-phenols of *Ajuga ciliata Bunge has* good anti-oxide activity.

#### 3.5.2 O_2_^-^· scavenging activity

As shown in Fig. 3B, as the poly-phenols concentration increased, the O_2_^-^· scavenging activity of PAB solution (15.21%–83.26%) rapidly changed. The results indicate that Vc has good O_2_^-^·scavenging activity. At low concentrations, the O_2_^-^· scavenging activity of PAB is less effective than vitamin C. However, at high concentrations, the O_2_^-^· clearance rate of PAB is superior to vitamin C. The results indicate that PAB exhibited a high O_2_^-^· scavenging activity.

#### 3.5.3 DPPH· scavenging activity

According to Fig. 3C, the DPPH· scavenging activity of PAB extracted by the UAE has shown that the capacity rose with concentration. When the concentration is in the range of 0.01–0.05 mg/mL, the DPPH· scavenging activity of PAB is slightly less than that of Vc. When the concentration was greater than 0.05 mg/mL, the antioxidant activity of PAB was the same as Vc, and the maximum inhibition percentage was 95.51%. In general, PAB exhibits significant DPPH· scavenging activity.

According to Fig. 3C, the DPPH• scavenging activity of PAB extracted from the UAE indicates that the capacity increases with increasing concentration. When the concentration is in the range of 0.01–0.05 mg/mL, the DPPH• scavenging activity of PAB is slightly lower than that of Vc. When the concentration was greater than 0.05 mg/mL, the antioxidant activity of PAB was the same as that of Vc, and the maximum inhibition percentage was 95.51%. Generally, PAB exhibits significant DPPH• scavenging activity.

### 3.6 Characterizes of poly-phenols

**Fig.4:**
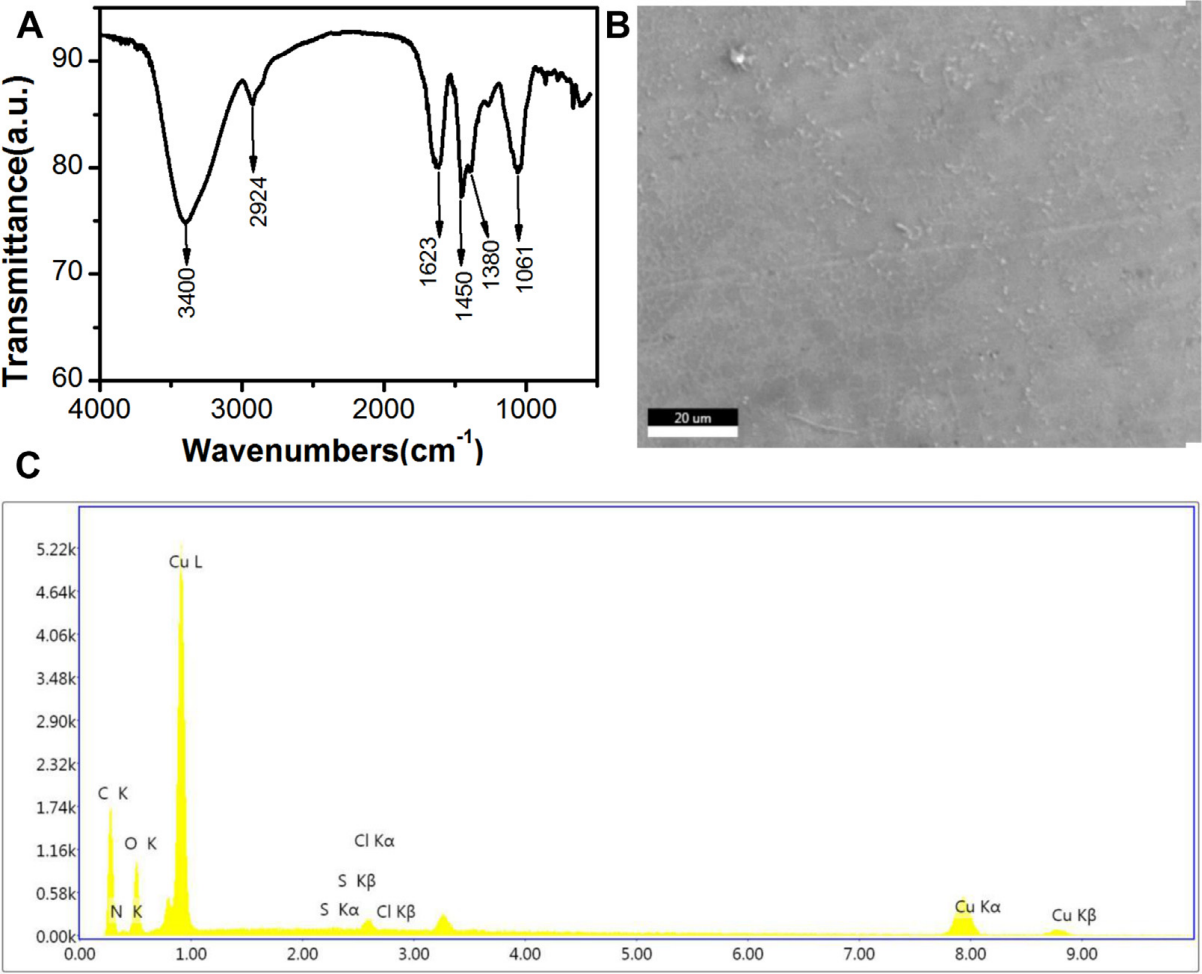
(A) FTIR spectrum of PAB, (B)SEM images of PAB (C)EDX spectrum of PAB.

Fourier infrared spectrum can reveal characteristics of compounds extracted from *Ajuga ciliata Bunge*. As present in Fig. 4A, the spectrum displays many absorption bands. The peak intensity at 3400 cm^−1^ is very strong, which provided direct evidence that a lot of hydroxyl (-OH) groups are present in PAB [43]. The peak intensity at 2924 cm^−1^ can attribute to methylene (–CH_2_–) roup and benzene ring [44,45]. The peak intensity at 1623 cm^−1^ can attribute to the stretching vibration of carboxyl ion (COO^−^), which provides direct evidence that the existence of carbonyl (C=O) groups in PAB [46,47]. The peak intensities at 1450 cm^−1^ and 1380 cm^−1^ are belonged to methyl (-CH_3_) group and the stretching vibration of C–O is shown in 1061 cm^−1^ [48].

Fig.4B shows the SEM images of the poly-phenols extracted from *Ajuga ciliata Bunge*. It can be seen that irregular shapes with diameters in the range 1∼3μm. This may be attributed to the fact that polyphenols are a complex mixture and therefore have no definite shape. The EDX spectrum of PAB reveals the presence of elements of carbon, nitrogen, oxygen, sulfur, chlorine. As can be seen from Fig.4C and Table 4, there is a small amount of nitrogen (2.08wt%), chlorine (1.02wt%) and sulfur (0.14wt%) in the extract. The results can be due to PAB may contain a small amount of impurities. Besides, the most abundant element in PAB is carbon (32.5wt%), followed by oxygen (10.69wt%). This is in agreement with the result of FTIR that there are a large number of oxygen-containing groups (C–O、COO^−^、C=O). Those results indicate that the compositions of PAB are a large number of phenolic compounds and a small amount of impurities.

**Table 4:** eZAF intelligent quantitative results of PAB.

Element	Weight %	Atom %	Net intensity	error
C K	32.95	61.94	140.7	0.01
N K	2.08	3.35	4.8	0.1
O K	10.69	15.09	85.7	0.02
S K	0.14	0.1	3	0.57
ClK	1.02	0.65	20.1	0.12
CuK	53.11	18.87	125.5	0.02

## 4 Conclusions

In the present study, poly-phenols are effectively extracted from plants named *Ajuga ciliata Bunge* by UAE method. To get the mutual influence of factors, RSM is used to obtain the optimal conditions as follows: EVF of 41%, LS ratio of 35:1 (mL/g), UTe of 60 °C and UTi of 50 min. Under optimal conditions, the experimental maximum yield of Y_max_ = 3.55166 mg/g. The spectra of Fourier infrared and EDX suggest phenolic compounds exist in the extracts. Besides, the antioxidant activity of PAB shows in the experiments of DPPH·, HO·, and O_2_^-^·is similar to that of Vc. It indicates that PAB using UAE method can be considered as a good natural antioxidant. This study shows that a natural antioxidant can be obtained by UAE from *Ajuga ciliata Bunge*, which may be used in pharmaceutical, food, feed, and other industries.

## Declarations

### Author contribution statement

Yanfei Zhang, Haitong Tan, Le Pan: Conceived and designed the experiments; Performed the experiments; Analyzed and interpreted the data; Contributed reagents, materials, analysis tools or data; Wrote the paper.

Yuchuan Zheng, Jinzhu Li: Analyzed and interpreted the data; Contributed reagents, materials, analysis tools or data; Wrote the paper.

### Funding statement

This work was supported by the National Natural Science Foundation of China (21401065), the Science Project of Education Department of Anhui Province (KJHS2018B04) and the Provincial Undergraduate Innovation Project (201710375012, 201810375092, 201810375112).

### Competing interest statement

The authors declare no conflict of interest.

### Additional information

No additional information is available for this paper.
